# Application of Phase-Reversal Fresnel Zone Plates for Improving The Elevation Resolution in Ultrasonic Testing with Phased Arrays

**DOI:** 10.3390/s19235080

**Published:** 2019-11-21

**Authors:** Dmitry O. Dolmatov, Daniel Tarrazó-Serrano, German A. Filippov, Igor V. Minin, Oleg V. Minin, Dmitry A. Sednev

**Affiliations:** 1 National Research Tomsk Polytechnic University, 30 Lenin Avenue, Tomsk 634050, Russia; ilippovga@tpu.ru (G.A.F.); prof.minin@gmail.com (I.V.M.); ovminin@tpu.ru (O.V.M.); sednev@tpu.ru (D.A.S.); 2 Centro de Tecnologías Físicas, Universitat Politècnica de València, Camí de Vera s/n, 46022 València, Spain; dtarrazo@fis.upv.es

**Keywords:** automated ultrasonic phased array inspections, 3D ultrasonic imaging, phased array resolution, Phase-Reversal Fresnel Zone Plate, ultrasound focusing, Sampling Phased Array

## Abstract

Currently, phased arrays have found wide application in ultrasonic nondestructive testing. Volumetric results provided by the inspections with linear phased arrays have low lateral resolution in the elevation direction of such probes. This fact complicates the defects characterization task. In this paper, we suggest the application of the Phase-Reversal Fresnel Zone Plate to increase the resolution of volumetric imaging with linear phased arrays. Application of such plates is aimed at ultrasonic focusing in the elevation plane whereas focusing on the active aperture plane is provided by the application of the Sampling Phased Array. Furthermore, the use of the Phase-Reversal Fresnel Zone Plate is advantageous due to the capability of its 3D printing and introduction to the existing automated testing systems avoiding making changes to the current software and hardware. The effectiveness of the plates was verified experimentally on the existing automated testing system. The obtained experimental results demonstrate that the application of the Phase-Reversal Fresnel Zone Plate allowed achieving the results of the higher resolution as well as improving the signal to noise ratio.

## 1. Introduction

**Figure 1:**
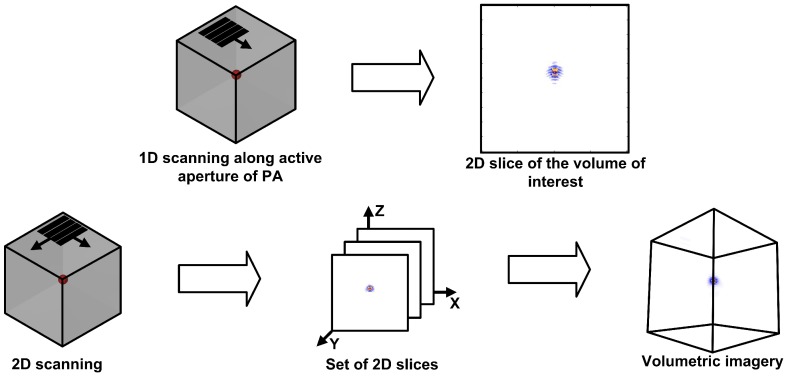
The principle of 3D imagery acquisition by the application of a linear phased array (PA).

In recent years, phased arrays (PA) have found wide applications in ultrasonic nondestructive testing. The advantages of such probes over the single element transducers are the ability to perform multiple inspections, provide higher sensitivity and results in the form of the imagery of the internal structure of testing objects [1]. Flaws in controlled objects are commonly volumetric and have arbitrary shape and orientation. Therefore, volumetric imaging is more preferable in order to effectively solve the defects characterization task. However, widely used nowadays linear PA is capable to provide only the 2D imagery of the internal structure of a testing object in a single position. Therefore, in automated testing systems with linear PA, 3D imagery are commonly obtained via the combination of 2D slices of the volume of interest. Such slices are obtained from the coherent summation of the imagery determined in each position of the PA during its motion along the active aperture of the PA (Figure 1).

However, such an approach is conditioned by low lateral resolution of the obtained imagery in the elevation direction of linear PA. It is due to the fact that in order to obtain high sensitivity of the inspections the PA elevation size is significantly greater than the width of a single element [2]. In order to increase the resolution of volumetric imagery obtained via imaging with PA, several approaches can be applied. The application of matrix PA instead of linear PA seems to be the most straightforward option. These transducers have an increased range of inspection angles and volumetric coverage in a single testing position. However, to ensure effective defect characterization a large number of elements are required to obtain the aperture of sufficient size when matrix PA is used. This conditions the necessity to process the large set of data for the imagery acquisition and demands the development of specific strategies for real-time imaging with matrix PA [3]. As a result, nowadays practical application of matrix PA is limited.

Furthermore, a combination of the rotatory and translatory motion of the linear PA during the scanning could be used for reducing the impact of the PA elevation on the resolution of obtained volumetric imagery [4]. According to such an approach, the medical imaging techniques based on Freehand scanning were developed [5]. For determining the position and orientation of PA in each position of the probe during the scanning it should be supplied with optical or electromagnetic sensors. Furthermore, such a technique requires the calibration procedure for obtaining the correct imagery.

Another approach aimed at increasing the resolution of the volumetric imaging with PA is the application of linear PA with acoustic lenses [6,7]. Acoustic lenses, as in other fields such as optics or electromagnetism, are the devices that allow energy beams modulation through different physical phenomena. Lenses represent great interest in different fields of science and engineering. We have found certain examples of lenses applications in the pharmaceutical field [8], sonochemistry [9] and construction [10]. The use of acoustic lenses in volumetric imaging with linear PA implies that the focusing in the elevation plane is provided by the acoustic lens whereas the focusing in the active aperture plane is obtained via the PA operation mode. The most evident advantage of the acoustic lens application is the capability to increase the quality of results provided by the existing testing systems while avoiding introducing significant changes to the equipment hardware and software. Results obtained via the application of acoustic lens are to possess not only the increased resolution but also high signal to noise ratio (SNR).

Several designs of acoustic lenses for the volumetric ultrasonic imaging with linear PA have been suggested recently. Brizuela et al. proposed the application of the special mask aimed at changing beam divergence in the elevation plane of PA [7]. The imaging approach by such mask utilization includes Phased Array Ultrasonic Technique (PAUT) application for the focusing in the active aperture plane, Synthetic Aperture Focusing Technique (SAFT) for the effective focusing in the elevation plane and increase of the SNR and Phase Coherence Imaging for reducing the side lobes and grating lobes on the final imagery. The effectiveness of the mentioned above imaging method was tested in the immersion and contact inspections. The quality of the obtained images was comparable with the results obtained by the equivalent matrix PA. However, the implementation of this approach in the existing testing system will require the changes in the equipment software in order to perform the proposed imaging procedure. Software modifications can be the most appropriate option at the research and development stage but in the case of commercial testing systems application, it could be impossible to make changes in the software. It is conditioned with the fact that commonly source code of the software of commercial testing systems is closed for users.

Raišutis et al. proposed the lens which provides fixed focusing in the elevation plane whereas in the active aperture plane focusing is obtained via the PAUT [6]. This lens is a plexiglass wedge with a concave gap filled with the water. The radius of concave was chosen in order to obtain the desired depth of the focusing inside the testing specimen in the elevation plane. The capability of the developed approach was tested by using thin specimens made of steel and metal foam composite. The tests demonstrated the increased resolution of the obtained results as well as higher efficiency of defect detection.

Unlike a concave or convex geometry lens, a flat lens like a Fresnel Zone Plate (FZP) has a planar surface. FZP has advantages in situations where size, weight, system complexity, and fabrication are important [11,12,13]. Traditionally, FZP is based on the application of a mask of transparent and opaque regions (Soret type). The current development in 3D printing technologies opens a new path in research in the FZP application. One of the new techniques proposed is Phase-Reversal FZP (PR-FZP). PR-FZP can be manufactured via the 3D printing and allows to improve the transmission and targeting using the phase change [14]. The particular configuration of PR-FZP can be determined with respect to the ultrasonic inspection conditions. However, despite the benefits of FZP applications in ultrasonic non-destructive testing, there is a lack of research in this field.

Thus, the objective of this work is to enhance the spatial resolution of ultrasonic imaging with linear PA via focusing on two planes. In the active aperture plane, the focusing is provided by the application of the Sampling Phased Array (SPA) technique [15] whereas the focusing in the elevation plane is provided by the FZP. Similar to the Total Focusing Method [16] and Inverse Wavefield Extrapolation [17] SPA is based on multi-element synthetic aperture principle and considered as ‘a gold standard’ in ultrasonic imaging with PA [18]. It is conditioned by the capability of these techniques to provide the results with high SNR and contrast resolution. SPA implies the data sampling via excitement of each element of the PA individually while the echo-signals are receipted by all elements of the PA (so-called Full Matrix Capture mode). Subsequently, the sampled ultrasonic data is processed by the application of the imaging algorithm based on SAFT in order to obtain the imagery of the internal structure of the controlled object.

## 2. Theory and Methodology

A zone plate is a flat lens that consists of several zones with different impedance for ultrasonic waves. Zones are spaced in order to provide the interference of the ultrasonic waves in the desired focus. In FZP each zone constitutes a Fresnel region, and between two consecutive regions, there is a π phase difference. A difference of π in the phase is equivalent to a difference of λ/2 in the propagation paths. Such an approach can be implemented in two ways. The first way implies the application of regions that are transparent and opaque for ultrasonic waves. This is the so-called Soret-type FZP. The second way is to perform the phase compensation in areas that contribute destructively to focusing in order to obtain the interference of the waves in the desired area (PR-FZP). In PR-FZP all the regions of the lens contribute constructively to the focal area, whereas in Soret-type FZP only the transparent zones contribute to the focal area. Therefore, PR-FZP has increased efficiency and intensity in comparison with Soret-type FZP.

Materials that are used for Soret-type FZP manufacturing should have either a high impedance contrast with the host medium or a high attenuation constant, which ensures a high reflection and a low transmission coefficient, respectively. Contrariwise the materials for PR-FZP should be chosen in order to obtain the maximum possible pressure through the phase-reversal regions. In this regard, polylactic acid (PLA) is a promising material for PR-FZP manufacturing due to appropriate acoustic properties and its widespread application in 3D printing. This means that PR-FZP with desired parameters can be developed and 3D printed in pretty fast fashion. In this regard, PLA is considered as a material of PR-FZP below.

In order to perform the focusing in the elevation plane of PA, it is necessary to apply one-dimensional FZP. The main FZP design parameters are focal length ($F_{L}$), defined as the location in the axial coordinate where the acoustic pressure field is focused; the Number of Fresnel zones (*N*) which includes sections with different ultrasound transparency and the working frequency ($f_{0}$). Once the design values have been set, the distances ($r_{n}$) of the implemented lens can be obtained using Equation (1), which depends on all the previous design parameters and is valid for the plane wave incidence [19].

(1) $$r_{n}=\sqrt{n\lambda F_{L}+{\left(\frac{n\lambda }{2}\right)}^{2}}\;n=1,2,3,\ldots ,N.$$

In our work, two hosts are considered, water layer and steel. Steel has the acoustic impedance higher than water. Therefore, this will cause a focus shift closer to the surface according to Snell’s law. Therefore, the $F_{L}$ design of the lens should be located much further than the desired $F_{L}$. This implies that the $F_{L}$ of the FZP will be modified as shown below,

(2) $$F_{L}=d+L\cdot \frac{c_{steel}}{c_{water}}.$$

**Figure 2:**
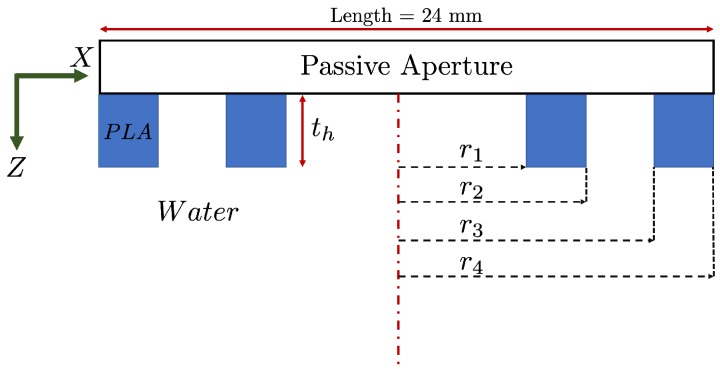
Phase-reversal (PR)-Fresnel zone plate (FZP) building scheme.

Once the dimensions of Fresnel zones are obtained it is possible to determine the thickness that accomplishes the PR-FZP condition [14]

(3) $$t_{h}=\frac{q}{k_{0}-k_{m}}\pi ,$$

where $k_{m}=2\pi /\lambda$ and $k_{0}=2\pi /\lambda_{0}$ are the wavenumbers of the lens material and the host medium, respectively. Also, *q* values are 1, 3, 5..., according to the phase-reversal regions having to be such that the phase difference introduced and compared to transparent regions is an odd multiple of π. The appropriately chosen thickness of PR-FZP provides the necessary phase correction. The phase increase is described as, $\Delta \theta =|k_{m}-k_{0}|t_{h}=\left(2n+1\right)\pi$. According to performed calculations, the most appropriate thickness for PR-FZP is 1.156 mm which provides the transmission factor of 97% for the given case. This value refers to the amount of energy that the lens is capable of transmitting to the first medium. Unlike a Soret-type FZP that blocks a portion of the transmitted energy, the PR-FZP allows a higher energy transmission of the transducer to the medium. Further, the Fresnel radii obtained for construction via Equation (1) are 5.97, 8.46, 10.38, 12.01 mm for $r_{1}$, $r_{2}$, $r_{3}$, and $r_{4}$ respectively. Figure 2 shows the scheme of the obtained PR-FZP.

### Numerical Models

**Figure 3:**
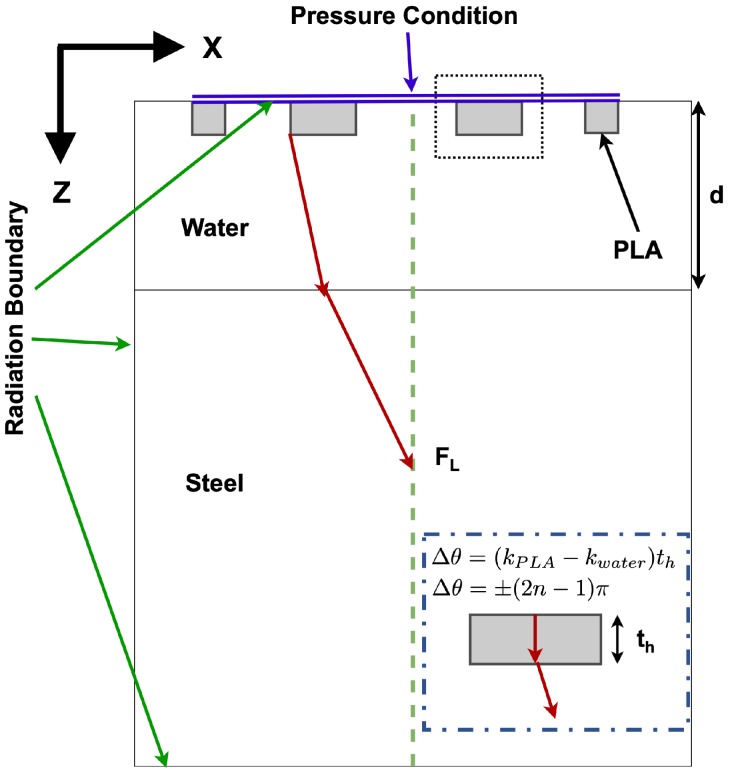
Finite elements method (FEM) Scheme of the implemented numerical model.

To determine the behavior of the FZP, a numerical model that replicates the behavior of the device has been used. The Finite Elements Method (FEM) allows us to create this type of model to replicate the characteristics of the problem. In this case, the commercial software COMSOL multiphysics has been used. Using this method, it has been possible to obtain the numerical solution that replicates the characteristics of the lens implemented and the interaction of the energy between both media to see the correct focalization of the lens. The acoustics module of COMSOL multiphysics allows us to obtain the solution to the Helmholtz Equation (4).

(4) $$\nabla \cdot \left(-\frac{1}{\rho }\left(\nabla p\right)\right)=\frac{k^{2}p}{\rho },$$

where, ρ is the density of the material, *f* the working frequency, *k* the number of wave defined by 2π/λ and *p* the pressure. In this work, there are two different media, water and steel. Figure 3 shows the conditions of the simulation. In this case, a two-dimensional model that simulates a cut of the elevation opening of the PA has been defined. To avoid internal reflexions, radiation boundaries have been selected to emulate the Sommerfeld condition. To define the acoustic impedance, the following sound velocity values (*c*) for water, steel and material of FZP (PLA) of 1500, 5900 and 2220 m·s${}^{-1}$ and the density values for water, steel and PLA of 1000, 7850 and 1240 kg·m${}^{-3}$ respectively, have been used. PA elevation has been considered as the pressure boundary contour condition. In order to consider real parameters, the Solid-Mechanics module is used as well for steel and PLA materials. Both materials have been considered isotropic and the linear elastic material contour condition has been used for each material. The Young modulus of 3.5 GPa and the 2.4 GPa shear module have been considered for PLA. Further, the shear-wave speed of 3230 m/s has been considered for steel. To obtain a coherent solution, the multi-physics module is used so that the contours are perfectly coupled. Mesh geometry selected was triangular. To avoid numerical dispersion, the minimum element size of $\lambda_{w}/16$ and the maximum element size of $\lambda_{w}/10$ have been chosen, where the $\lambda_{w}$ is the wavelength in water.

**Figure 4:**
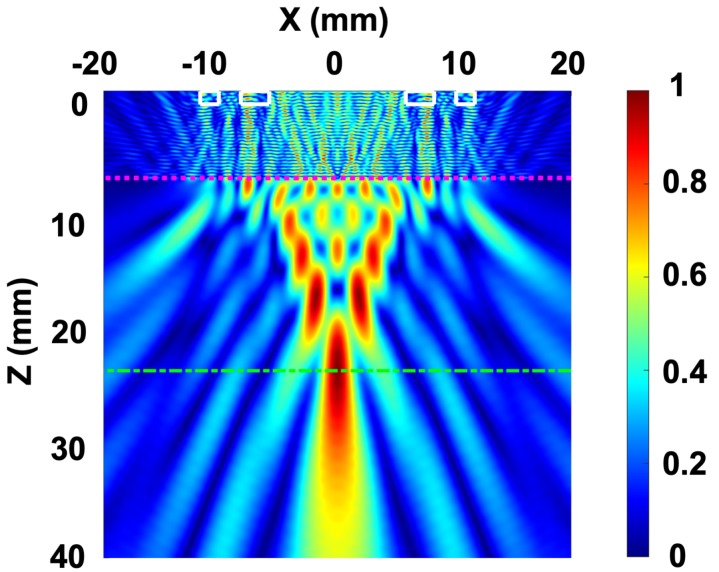
Numerical results of the normalized acoustic pressure with a 6.5 mm water layer.

Figure 4 shows the result with FEM model taking into account the water layer of 6.5 mm (from the edge of the plate) and the designed $F_{L}$ of 93.03 mm that is equivalent to the 17.1 mm focal spot inside the steel. It can be seen that compared to the length of the element of PA (24 mm), a relatively small focus size is obtained. Full width half maximum (FWHM) value of 2.63 mm and full length half maximum value of 38.12 mm are achieved. FWHM is directly related to lateral resolution. This reduced value allows for correct detection of relatively small flaws.

## 3. Experimental Set-Up

**Figure 5:**
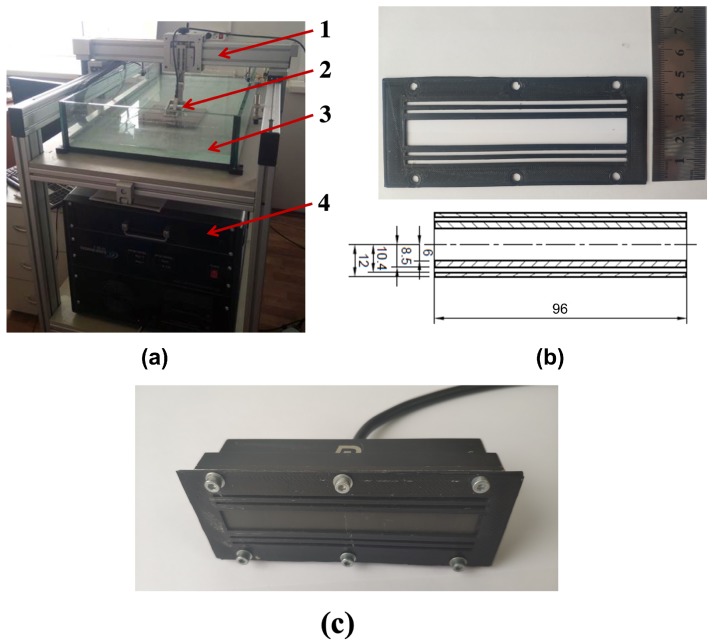
Photo of (**a**) Idealsystem3D experimental set-up, where 1 is the ultrasonic scanner, 2 PA, 3 immersion bath and 4 electronic unit. Photo and scheme of (**b**) manufactured PR-FZP. Photo of the lens (**c**) mounted in the PA.

The evaluation of the lens effectiveness was conducted via the Idealsystem3D (I-deal technologies GmbH Germany) application (Figure 5a). This is the ultrasonic inspection and analysis system which consists of several components including an electronic unit with an integrated PA system and a motion control unit that has a two-axis manipulator for the scanning the inspection objects. Idealsystem3D software has been used for data processing and inspection of the results. Idealsystem3D provides the testing results in the form of 3D imagery obtained via the combination of 2D slices of the volume of interest [20]. The acquisition of 2D slices is performed via the application of Sampling Phased Array technique.

Linear PA Doppler 2.5L64-1.5x24 was used in experiments. The probe consists of 64 elements with a central frequency of 2.5 MHz. the length of each element of PA is 24 mm and width is 1.1 mm. The pitch size of the PA is 1.5 mm. The length of the active aperture is 96 mm and length of passive aperture (elevation) is 24 mm.

Applying the construction Equation (1), the different dimensions of Fresnel zones are obtained. The Fresnel zones that match the PA size limitation are implemented. The FZP with the parameters obtained via the FEM modeling was printed using a commercial 3D printer and PLA filament (Figure 5b). The construction dimensions in millimeters are shown. Finally, in Figure 5c, it is shown how the lens is placed on the PA.

**Figure 6:**
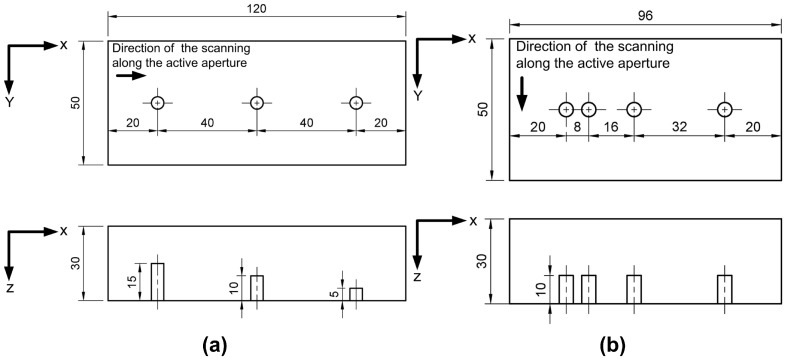
Location of the defects in blocks (**a**) A, B and C and (**b**) block D.

Experimental verification of the developed acoustic lens was carried out via inspection of the testing blocks with flat bottom holes. The experimental verification comprised two stages. During the first stage, the impact of the size of the flaws on the imaging with the acoustic lens was studied. For this purpose, three steel blocks with the same location of the flaws were used (Figure 6a). Each block contained flat bottom holes with specific diameters: 5 mm (block A), 3 mm (block B) and 2 mm (block C). During the second stage, the lens performance in the case of closely-spaced flaws was evaluated. For this case block D with flat bottom holes with diameter 5 mm was used (Figure 6b). In this case, the scanning path was chosen so that the defects were located along the elevation of PA. This case can be considered as the most challenging one for the defects characterization with linear PA application.

In order to obtain the volumetric imagery, the two-dimensional scanning of the blocks was performed in experiments. The scanning step along both axes was 1 mm. The ultrasonic waves were generated by using 200 V tone burst excitation signal with a duration of 1.3 microseconds In all positions of the scanning path, the ultrasonic data was sampled in the Full Matrix Capture mode. Due to the limitation of IdealSystem 3D only 16 electronic channels can be applied for ultrasonic data acquisition. This means that only 16 elements of the PA were active and were able to emit ultrasonic waves and acquire echo-signals for their further processing in order to obtain the imagery of the testing objects.

## 4. Results and Discussion

**Figure 7:**
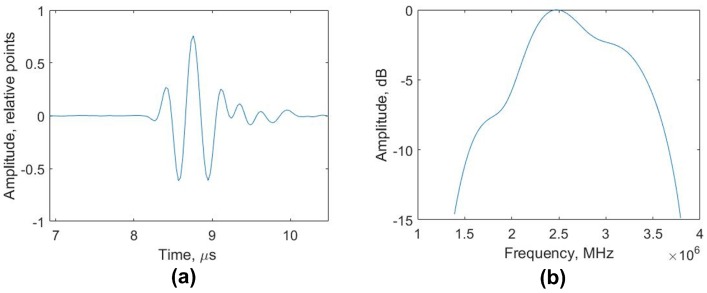
The pulse-echo response of the single PA element from the surface of the testing object (depth 6.5 mm) (**a**) the magnitude of the pulse spectrum (**b**).

First of all, it is necessary to confirm that the PA operates at the central frequency for which the PR-FZP was designed. This can be done via the analyzing of raw signals. Figure 7 shows representative pulse-echo signal which is the result of ultrasonic wave reflection from the surface of the testing block. The bandwidth of the signal was 60% and the measured central frequency was 2.47 MHz.

**Figure 8:**
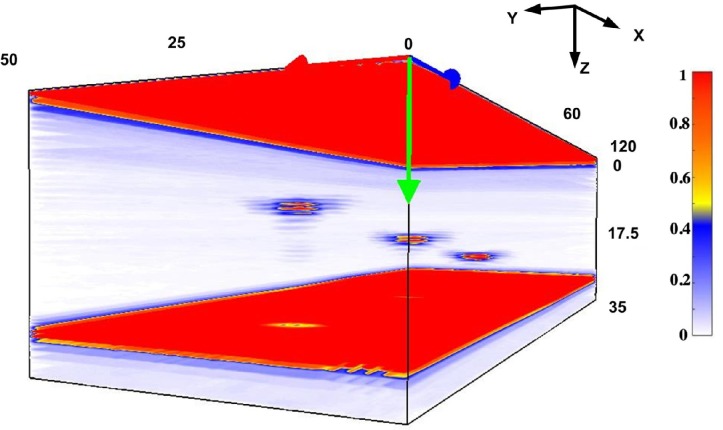
Example of obtained volumetric imagery.

The inspection and analysis system provides the experimental results in the form of 3D imagery (Figure 8). The capability of various zones of the internal structure of the controlled object to reflect the ultrasonic waves is denoted by the color according to the color bar of imagery. The areas where the ultrasound reflection occurs in the greatest way are the surface, backwall, and flaws. In order to compare the results and find out if the PR-FZP is capable of improving the lateral resolution in elevation plane, two-dimensional projections of volumetric imagery are required.

**Figure 9:**
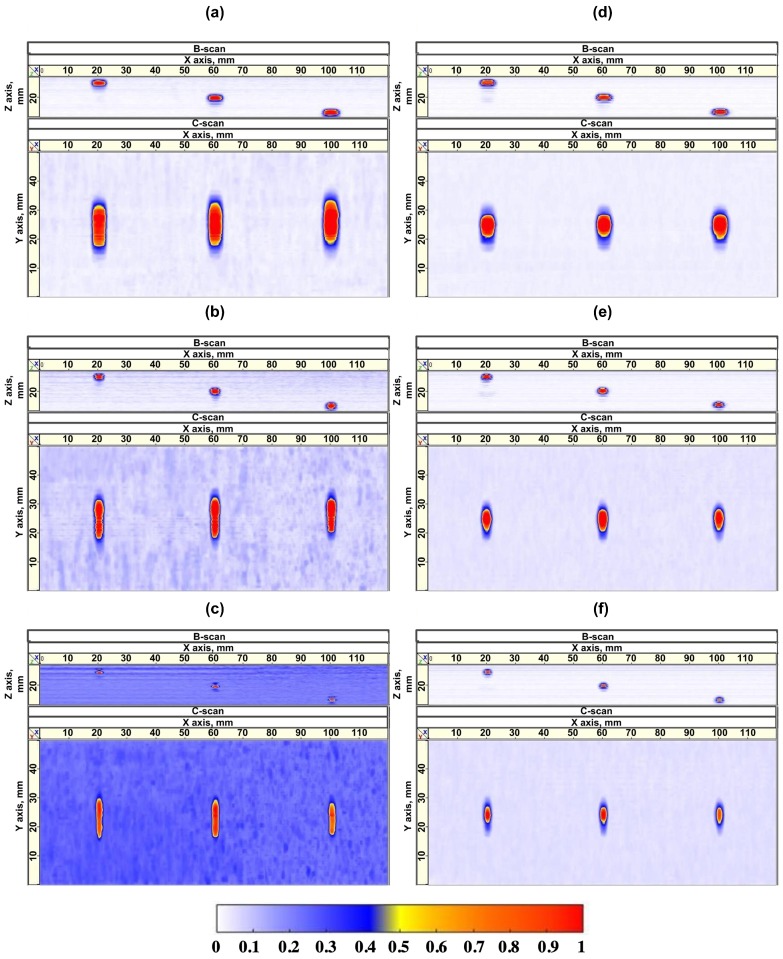
Two projections of obtained of volumetric result for blocks (**a**) A, (**b**) B, and (**c**) C without lens and blocks (**d**) A, (**e**) B, and (**f**) C using PR-FZP.

Figure 9 presents the imagery projections (B and C-scans) from the volumetric results. In the Figure 9a–c the projections of the volumetric results for blocks A, B, C without the PR-FZP application are presented. The same data with the PR-FZP application is presented in Figure 9d–f. All the results have the same color range.

**Figure 10:**
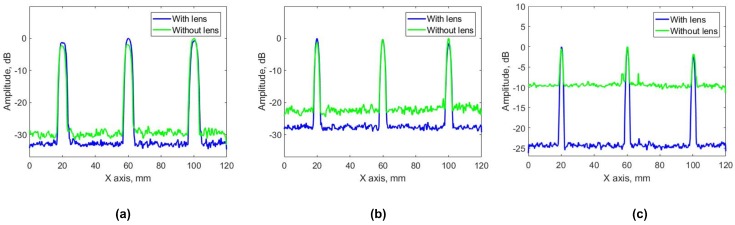
Lateral profile of results obtained for block (**a**) A, (**b**) B, and (**c**) C on the X-axis.

**Figure 11:**
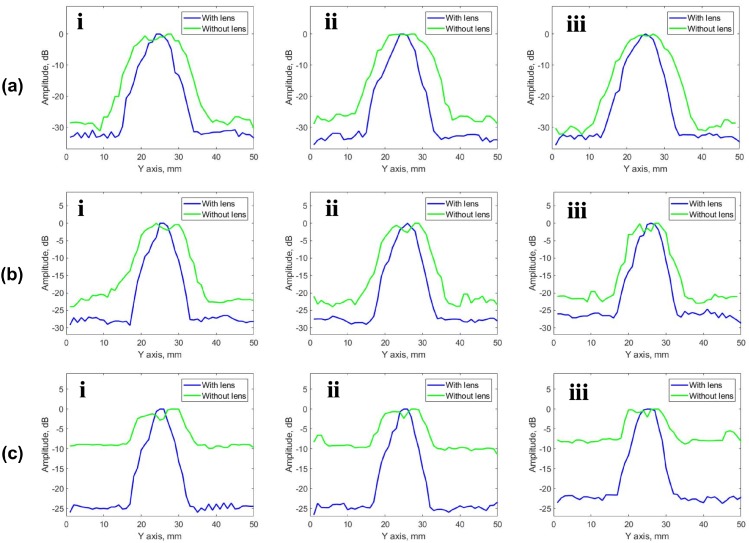
Lateral profiles of result obtained for blocks (**a**) A, (**b**) B, and (**c**) C, on the Y axis for each of the flaw: i—depth of the drilling 15 mm; ii—depth of the drilling 20 mm; iii—depth of the drilling 25 mm.

Two phenomena can be observed in each pair of subfigures (a and d, b and e, c and f) for the different flaw sizes 5, 3 and 2 mm, respectively. First, when PR-FZP is not used the imagery of circular flaws is elongated along the elevation of PA. This reveals the lack of lateral resolution of imagery. Once the lens is introduced, the shape of flaws obtained in the imagery is close to circular. Further, the obtained results demonstrate that SNR of results obtained with PR-FZP application is higher than the results without its utilization. This can be verified numerically in Figure 10 and Figure 11 by comparing lateral profiles. In order to compare the resolution of the obtained results as well as SNR all the obtained results were exported to Matlab R 2016b for the acquisition of their lateral profiles. The lateral profiles (on both axis) for different blocks in the case of lens application and without it are presented in Figure 10 for X-axis and Figure 11 for Y-axis lateral profiles, respectively.

Taking the average values of the noise background for each block, the improvement of SNR can be obtained for each case by comparing it with and without PR-FZP. SNR improvement obtained for each case is 3, 6 and 15 dB for blocks A, B, and C, respectively.

The results demonstrate the efficiency of the PR-FZP application. The imagery with the improved lateral resolution in elevation plane was obtained. However, the imagery has a similar resolution along the active aperture direction. Furthermore, the imagery obtained via lens application has higher SNR in comparison with the cases where imagery acquisition was performed without lens application. The improvement in SNR becomes more significant when smaller flaws are visualized. This fact can serve as evidence of increased sensitivity with lens application.

**Figure 12:**
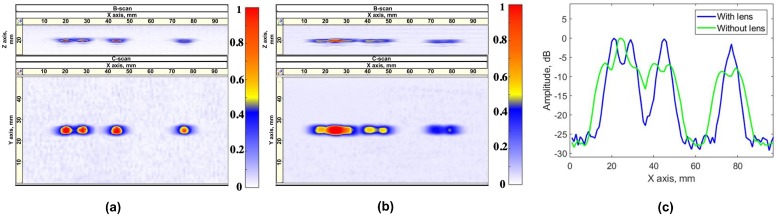
Two projections of obtained volumetric result for the block D, (**a**) with PR-FZP, and (**b**) without PR-FZP. (**c**) Lateral profile of result obtained for block D on the X axis.

During the next stage, the PR-FZP performance was evaluated locating the close-spaced defects along with the elevation of PA. The two projections of volumetric results in the case of the lens are presented in Figure 12a, whereas the same results for the case when the lens has not been applied are presented in Figure 12b. The lateral profiles of the obtained results are presented in Figure 12c.

According to the obtained results for the given case, the defect characterization task can be solved more effectively by the application of PR-FZP. This conclusion could be made according to the obtained lateral profile of the result. On this profile, there are the peaks that correspond to each of the flaws. Defects are considered to be resolved if the amplitude between their peaks decreases by at least −6 dB with respect to the peak maximum with lower amplitude [21]. Following this principle, the lens was allowed to detect all the flaws separately for the given case whereas conventional ultrasonic imaging with PA provided the imagery where two closest flaws appeared as one.

## 5. Conclusions

In this article, the PR-FZP application for improvement of the resolution of linear PA inspections was considered. The utilization of such a lens is aimed at focusing on the elevation plane whereas focusing on the active aperture plane is obtained via the PA operation mode. Such dual-focusing mode allows obtaining the high-resolution volumetric results in the case of imaging with linear PA. Furthermore, PR-FZP can be introduced to the existing automated testing systems avoiding making the changes in software and hardware of this equipment. Another advantage of the PR-FZP application is the capability of its manufacturing using commercial 3D printers.

In this work, the parameters of the developed PR-FZP were determined with respect to the conditions of the planned experiment. The effectiveness of the PR-FZP design was verified via the FEM modeling. Subsequently, the acoustic lens was manufactured on the 3D printer. During the experimental verification, the developed PR-FZP was used in the existing automated testing system (IdealSystem3D) via its mounting on PA. During the experiments, the results obtained via the lens application were compared to the results when the PR-FZP was not used. During the experimental verification the testing specimens containing the flaws with different diameters, location and depth were used.

There are several limitations using this technique. First, the relatively small size of the passive aperture limits the number of Fresnel zones which limited the resolution of a lens. Second, there may be certain inaccuracy due to the precision of the 3D printing. Both limitations can be solved using first, higher frequency circular transducers and second, with the new 3D printing systems that are currently being released. The third limitation is conditioned by the application of toneburst signals with a Fresnel lens. Fresnel lenses are more effective when a continuous-wave is used. If the signal used to generate the image is not long enough, there will be inconsistencies in focusing. Accordingly, there will be a compromise between coherence of focus, linked to the resolution of the imaging system and the limitation of the imaging system. Furthermore, the imaging with PR-FZP in the single position of PA is associated with a relatively high level of side lobes and degradation of axial resolution which increases with the increment of the angle between the imaging point and central line of the probe. The impact of both of these factors is minimized via the coherent summation of the imagery obtained in various positions of PA during the scanning.

Despite the pointed issues, obtained experimental results demonstrate the efficiency of PR-FZP to improve the lateral resolution of volumetric PA imaging. Focusing properties of PR-FZP can be defined by the appropriate selection of the lens design taking into account the conditions of the PA inspection (e.g., desired depth of the focusing). As a result, the appropriate acoustic lens design can be 3D printed. In all cases, the PR-FZP application allowed obtaining the results with higher lateral resolution and SNR. Thus, the versatility of PR-FZP, as well as availability, makes the application of PR-FZP a promising approach in the field of PA inspections.
